# Temperature Effect of Water Coagulation Bath on Chitin Fiber Prepared through Wet-Spinning Process

**DOI:** 10.3390/polym13121909

**Published:** 2021-06-08

**Authors:** Khoa Dang Nguyen

**Affiliations:** Faculty of Technology, Van Lang University, 45 Nguyen Khac Nhu, Co Giang Ward, District 1, Ho Chi Minh 700000, Vietnam; khoa.nd@vlu.edu.vn

**Keywords:** biopolymer, chitin, crab shell waste, fiber, phase inversion, wet spinning

## Abstract

Chitin was chemically extracted from crab shell waste and dissolved in *N*,*N*-dimethyl acetamine/5% lithium chloride (DMAc/5% LiCl) at room temperature to obtain 1% and 2% concentrations of chitin solution. Chitin fibers were prepared by phase inversion at different temperatures of water coagulation bath at 5, 20, and 60 °C. The deconvolution of FTIR spectra indicated that the area portion of the intermolecular hydrogen bonding NH^…^OC increased at 60 °C due to the higher density of the chitin segment in the fiber. As a result, scanning electron microscope (SEM) measurement suggests that a denser structure of the chitin fiber was observed when the temperature of the coagulation bath increased. In addition, the resultant chitin fibers generated better mechanical properties relative to the amount of chitin concentration and temperature. At 2% of chitin solution, the tensile strength significantly increased from 80 to 182 MPa for the fiber obtained at temperatures of 5 and 60 °C of the water coagulation bath, respectively. Meanwhile, the water content in the fiber significantly decreased from 1101% to 335%. This green synthesis route has high potential for the fabrication of the fiber as future material of interest for biomedical application.

## 1. Introduction

**Figure 1:**
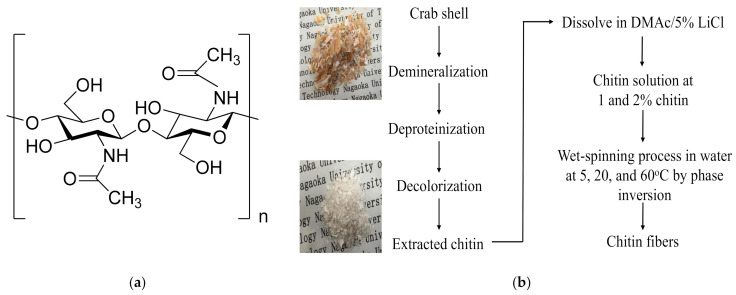
Chemical structure of (**a**) chitin and (**b**) the preparation procedure of chitin extracted from crab shell and chitin fiber fabrication.

It is known that one-dimensional (1D) polymeric fiber has been found to be attractive owing to its high surface-to-volume ratio. Because of this property, 1D fibers exhibit favorable mass transfer, offering distinct benefits for different applications [1]. So far, 1D fiber can be prepared by several technics depending on the desired diameter of the obtained fiber, such as electrospinning [2], microfluidic spinning [3], extrusion [4], and wet spinning [5]. In previous research studies, zeolite–polymer composite fiber has already been developed with polyethersulfone (PES) as scaffold for the effective adsorption of radioactive cesium in Fukushima [6] and heavy metal ions [7] through the wet-spinning process. In this technique, a polymer solution is spun into a coagulation bath for the immersion precipitation process. After the given period of time, the formation of fiber occurs due to the exchange of solvent and nonsolvent. Therefore, in the wet-spinning process, various parameters cause effects on the utmost structure of the spun fiber, including solvent/nonsolvent pairs, polymer concentration, temperature of the coagulation bath, and so forth [8]. For example, Kitagawa et al. reported that the poly-*p*-phenylenebenzobisoxazole (PBO) fiber prepared with 85 °C water vapor coagulation bath gained higher strength and modulus compared with water vapor [9]. Ji et al. suggested improvement in the physical properties of the as-spun polyacrylonitrile (PAN) fiber, such as crystallinity, orientation, and tenacity [10]. When the PAN fiber was prepared at high-temperature coagulation bath, the cross section deviated a little from a circular form, with the denser structure that was obtained [11]. However, most of the mentioned studies showed that synthetic polymers have been utilized to fabricate the fiber. Owing to the burgeoning interest in environmental issues, there is now a greater emphasis on green routes for inexpensive and environmentally benign materials as biomass-regenerated polymer. Till now, the potential applications of biopolymer-based material are gaining much attention owing to the favorable properties of the natural-origin fiber, such as hydrophilicity, biocompatibility, biodegradation, and nontoxicity [12,13]. In accordance with this purpose, in the present study, biopolymer-based fiber was fabricated using phase inversion in water as coagulant through the wet-spinning process. Among biopolymer materials, chitin is the second most abundant polysaccharide, which is widely distributed in the structural component of crustacean shells, which are waste products of seafood processing industries. The chemical structure of chitin is composed of β-(1 → 4)-*N*-acetyl-d-glucosamine units and is known for its several advantages, such as biocompatibility, biodegradability, and low toxicity, as shown in Figure 1a. Reported studies have already shown that biopolymer such as chitin is a potential material in the drug delivery system for medicine [14] and adsorbent for the removal of heavy metal ions in wastewater [15]. In addition, research on the preparation of chitin fiber through the wet-spinning process has not been reported elsewhere regarding the effect of the temperature of the water coagulation bath. Tokura et al. produced chitin fibers by dissolving chitin in various different solvents. However, only chitin solution dissolved in formic acid/dichloroacetic acid and isopropyl ether prepared by freeze-thawing method was proven to be spinnable. It is known that formic acid is considered a toxic solvent and not environmentally friendly. In addition, the involved process was supported by two consecutive coagulation baths containing ethyl acetate and cold water at 12–14 °C [16]. Contrarily, most of the studies focused on the preparation of the blended chitin fiber. For example, Zhang et al. reported the preparation of blended cellulose/chitin fiber through the wet-spinning process. Chitin was first dissolved in NaOH/thiourea/urea aqueous solution and then spun in the coagulation bath composed of H_2_SO_4_ and Na_2_SO_4_ aqueous solution [17]. In both of the mentioned studies, the preparation of chitin solution was complicated, using several solvents and prepared at a low temperature with a repeated process. Additionally, the fabrication of chitin fiber through the spinning method was performed using a two-step coagulation bath process with different washing solvents. Nevertheless, the morphology structure of the fiber was observed only on the surface area. Ota et al. suggested that chitin/cellulose-blended fiber was successfully prepared through the dry- and wet-spinning process in water coagulation bath at 95 °C [18]. Moreover, the mechanism of the fiber formation as regards the temperature effect of coagulation solvent is still unclear. Due to this, the aim of the present research is to study the temperature effect of coagulation bath and the concentration of polymer in the formation of chitin fiber. Here, chitin was chemically extracted from crab shell waste due to increasing interest in the utilization of renewable feedstocks for chemicals. The reuse of waste or by-products can increase economic value and environmental benefits and better highlight sustainability [19,20,21]. After that, the extracted chitin was dissolved in *N*,*N*-dimethyl acetamine/5% lithium chloride (DMAc/5% LiCl), without leading to the depolymerization of the biopolymer [22,23]. It is indicated that an amount of 5% LiCl in DMAc generates better properties than others [24]. Herein follows a description of the method adopted for fiber preparation via the wet-spinning process and fiber characterizations. The present paper includes evidence related to the temperature effect of coagulation bath on the properties of the prepared chitin fiber. The results suggest that the physical properties of the obtained fibers significantly increased in mechanical strength at a high temperature. In addition, the DMAc/LiCl solvent used in the preparation process has been reported to have good biocompatibility with the NIH3T3 fibroblast [12,25,26]. This green synthesis route has high potential for the fabrication of the fiber as future material of interest for biomedical application.

## 2. Materials and Methods

### 2.1. Materials

Crab shells were collected from red snow crabs (genus *Chionoecetes*) in Teradomari, Niigata, Japan. All chemicals used in the preparation of chitin were of analytical grade. Hydrochloric acid (HCl), sodium hydroxide (NaOH), potassium hydroxide (KOH), *N*,*N*-dimethyl acetamine (DMAc), lithium chloride (LiCl), and ethanol were products of Nacalai Tesque, Inc. (Tokyo, Japan). Prior to the use of DMAc, the solvent was stored with KOH for over 5 days, and LiCl was dried in vacuum oven at 80 °C for 24 h to remove trace of moisture.

### 2.2. Extraction of Chitin from Crab Shell Waste

In the present work, chitin was extracted from crab shells by chemical method, as shown in Figure 1b, with some modifications [25]. Briefly, in the demineralization, 10 g of crab shells was treated with 300 mL of 1 M HCl aqueous solution at room temperature for 24 h. Then, deproteinization was carried out in 300 mL of 10% NaOH aqueous solution at 90 °C for 5 h. Next, 300 mL of ethanol solution was added, and the proteinized crab shells were bleached at 60 °C for 6 h. The solid part was filtered off and washed with distilled water to neutral pH. The extracted chitin was dried in the vacuum chamber for 24 h at room temperature. The yield of the purified chitin was calculated by Equation (1):

Yield (%) = (m_0_ − m) × 100/m_0_ (1)

where m_0_ and m are the weights of the crab shells and the extracted chitin (g), respectively.

### 2.3. Preparation of Chitin Fiber through Wet-Spinning Process

**Figure 2:**
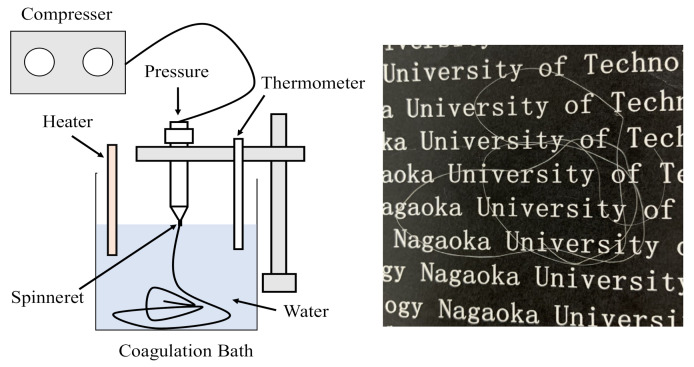
Formation of chitin hydrogel fiber obtained at different temperatures of the coagulation bath via the wet-spinning process and chitin hydrogel fiber.

Chitin was dissolved in DMAc with 5% LiCl for 5 days (wt/wt%). After being centrifuged at 9000 rpm for 30 min, chitin solutions were obtained at various concentrations as 1% and 2% chitin, which were denoted as C1 and C2, respectively. The formation of chitin fibers is presented in Figure 2. These chitin solutions were extruded through a cylindrical needle (Unicon Control Company, UNP-20, Niigata, Japan) with a 0.6 mm needle-hole diameter with 0.02 MPa of air pressure. The extruded solutions containing the extructor (Unicon Control Company, AJ-10, SB50, Niigata, Japan) were dropped into a water coagulation bath (250×250×150 mm^3^) for the phase inversion process. Here, the temperature of the water coagulation bath was varied at 5, 20, and 60 °C by a heater (IC Control, SCH-900SC, Monotaro, Hyogo, Japan). The related fibers were denoted as C1-20 for the samples containing 1% chitin and obtained at 20 °C. These chitin fibers were immersed in the coagulation bath for 24 h and then washed in distilled water to remove residual solvent before measurements.

### 2.4. Characterization of Chitin Solution and Chitin Fiber

#### 2.4.1. Viscosity

For the characteristics of the chitin solutions and the chitin fibers, various experiments were conducted to determine the effect of the temperature of the water coagulation bath. The viscosity of the obtained chitin solutions was determined at different temperatures, from 5 to 60 °C, with a fixed shear rate of 1 s^−1^ by a rheometer (Physica MCR 301, Anton Paar).

#### 2.4.2. Gel Permeation Chromatography (GPC)

GPC was used to determine the molecular weight distribution of the prepared chitin. The GPC system consisted of an online degasser (DGU-20A, Shimadzu, Japan), refractive index (RI) detector (RID-10A, Shimadzu, Japan), high-pressure pump (LC-20AD, Shimadzu, Japan), manual injector (7725i, Rheodyne), and GPC column (KD-806M, Shodex). Chromatogram was recorded by a chromatopac integrator (C-R8A, Shimadzu, Japan). The column temperature and the RI detector cell remained at 50 and 40 °C, respectively. As eluent, 1 g of LiCl in 99 g of DMAc solution was used. The GPC system was calibrated with narrow distribution polystyrene standards (TSK standard polystyrene, Tosoh, Yamaguchi, Japan). Then, 0.1 g of the extracted chitin was dissolved in 9.9 g of DMAc/8% LiCl solution. Finally, the sample solutions were diluted with DMAc to obtain 0.1% chitin in DMAc/1% LiCl solution. The injection volume was 100 μL. Before that, sample solutions were filtered using a poly(tetrafluoroethylene) (PTFE) disposable membrane filter with a 0.45 μm pore size.

#### 2.4.3. Fourier-Transform Infrared Spectroscopy (FTIR)

FTIR spectrum was recorded with a Jasco FTIR/4100 spectrometer by grinding dried samples with potassium bromide (KBr) in the transmittance model. The spectra were taken from 4000 to 500 cm^−1^ wavenumber with a resolution of 2 cm^−1^. The degree of acetylation (DA) of the treated chitin was calculated according to the method proposed by Moore and Roberts [26] using Equation (2):

(2) $$\mathrm{DA}\;(\%)=(A_{1650}/A_{3450})\;\times 100/1.3$$

where *A*_1650_ and *A*_3450_ refer to the absorbance of peak at 1650 and 3450 cm^−1^, which are related to the band of amide I and hydroxyl group, respectively, in the FTIR spectra. The deconvolution of the IR spectra in the range of 3700–3000 cm^−1^ was carried out. The center of the fixed peaks obtained from the spectrum date was decomposed into Gaussian components. The peal centers and the curve were then fitted using OriginPro 8.5.

#### 2.4.4. Size Measurement

A phase-contrast inverted light microscope (Eclipse TS100-F, Nikon, Japan) was used at ambient temperature to measure the average diameters of the prepared chitin fiber.

#### 2.4.5. Morphology Observation

Scanning electron microscopy (SEM) was used for the morphology of the cross section of the prepared fibers. The chitin fibers were dehydrated in 50% ethanol aqueous solution for 3 h, followed by soaking in ethanol solution for 2 h. After that, the dehydrated chitin fibers were freeze-dried for 24 h. For the measurement, all of the samples were fractured in liquid nitrogen. Then, gold sputtering was carried out for the formation of a conductive layer (JSM-IT300, JEOL, Japan).

#### 2.4.6. Diffusivity Experiment

The diffusivity of the water in DMAc and DMAc in water was determined at different temperatures to clarify the tendency of the diffusion in the formation of the chitin fiber [8]. Here, the diffusivity of one component in an organic solvent, such as DMAc, was calculated by Equation (3):

(3) $$\frac{D_{AB}^{0}\times \mu_{B}}{T}=8.52\times 10^{-8}\times [1.40\times (\frac{V_{bB}}{V_{bA}}{)}^{\frac{1}{3}}+\frac{V_{bB}}{V_{bA}}]$$

In the case of diffusion in an aqueous solution, the value of the diffusivity was calculated by Equation (4):

(4) $$D_{AB}^{0}=\frac{14.0\times 10^{-5}}{\mu_{wT}^{1.1}V_{bA}^{0.6}}$$

where $D_{AB}^{0}$ is the diffusivity of A molecule in B solvent (cm^2^/s), $\mu_{B}$ is the solvent viscosity (cP), T is the temperature (K), and $V_{b}$ is the molar volume of the solvent at its normal boiling temperature (cm^3^/g).

#### 2.4.7. Water Content

The water content (WC) of the chitin fibers was measured at room temperature by immersing 50 mm×0.1 mm of the dry fibers in distilled water for 24 h to reach the equilibrium condition. After that, the fibers were quickly removed and wiped with tissue to eliminate the unbounded water. The value of WC was calculated for each sample by Equation (5):

(5) $$\mathrm{WC}\;(\%)=\;\left(m-m_{0}\right)\times 100/m_{0}\;$$

where m_0_ is the dry weight and m is the weight of the fiber in distilled water.

#### 2.4.8. Mechanical Properties

The mechanical properties of the chitin fibers were evaluated for tensile strength and viscoelasticity. In the tensile strength test, the experiment was carried out by using LTS-500N-S20 (Minebea, Japan) with an operating head load of 50 N at 23 °C and 50% RH. The dry fibers (50 mm×0.1 mm) were then placed between the grips of the testing machine. The initial length was 20 mm, and the speed of testing was 2 mm/min till the sample was broken. The values of tensile strength and elongation were calculated using Equations (6) and (7):

Tensile strength (N/mm^2^) = maximum load/cross-sectional area (6)

(7) Elongation (%)=(elongation at rupture/initial gauge length)×100

## 3. Results and Discussion

### 3.1. The Properties of the Prepared Chitin

The extracted chitin was noncolored after the chemical treatment, as shown in Figure 1b. The yield of chitin after purification from carb shells was gained at about 33.5%, and the value of DA for the extracted chitin was 74.6% from the FTIR spectrum. The molecular weight distribution of the prepared chitin was 5.4 × 10^5^ g/mol.

### 3.2. Characteristics of the Chitin Solution and Chitin Fiber Obtained at Different Temperatures of the Water Coagulation Bath

**Figure 3:**
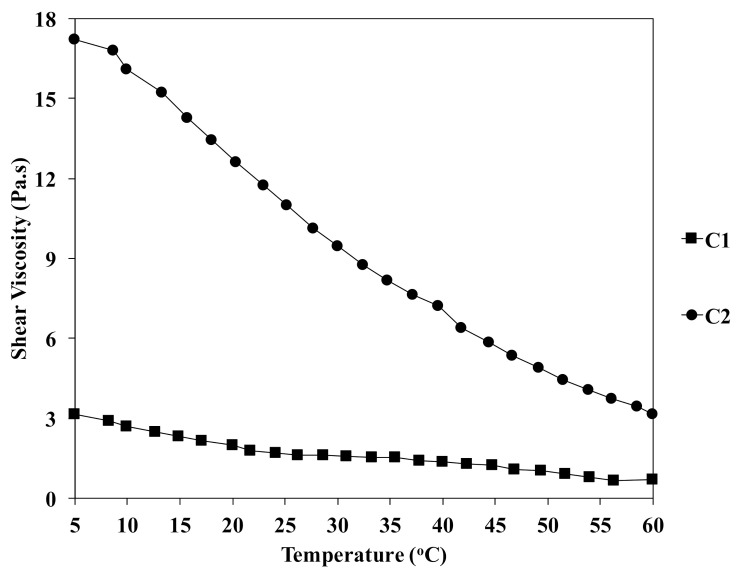
Effect of temperature on the viscosity of chitin solutions at different concentrations.

Figure 3 shows the shear viscosity of the chitin solution at different concentrations as a function of temperature from 5 to 60 °C. It was observed that the shear viscosity increased gradually with the increment of the concentration of chitin. For example, the values of shear viscosity were 3.1 and 17.3 Pa.s for C1-5 and C2-5, respectively. The relationship between the shear viscosity of chitin solution and temperature also was recorded. In the case of C2, the shear viscosity was dramatically decreased to 12.6 and 3.2 at 20 and 60 °C. This could be due to the evaporation of the volatile organic solvent at a high temperature. Additionally, increase in temperature reduced the cohesive forces between the molecules of the liquid, which could accelerate the gelation process during the fiber formation. The value of the viscosity of chitin solution in DMAc/LiCl is listed in Table 1.

In Figure 2, the dry chitin fiber was translucent after vacuum drying at 50 °C. As listed in Table 1, the diameters of the related fibers obtained at various temperatures of the coagulation bath and polymer concentrations were influenced. For instance, in the preparation of chitin fiber at 20 °C, the diameter was increased from 69 to 127 µm for C1 and C2, respectively. However, when the chitin concentration was used at 2% for the preparation of fibers, the diameters were somehow changed to 108, 127, and 102 µm at 5, 20, and 60 °C.

**Figure 4:**
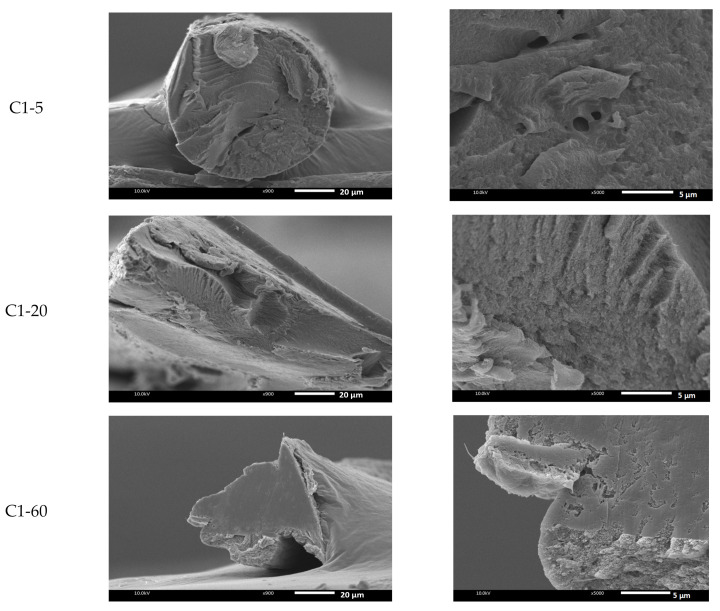

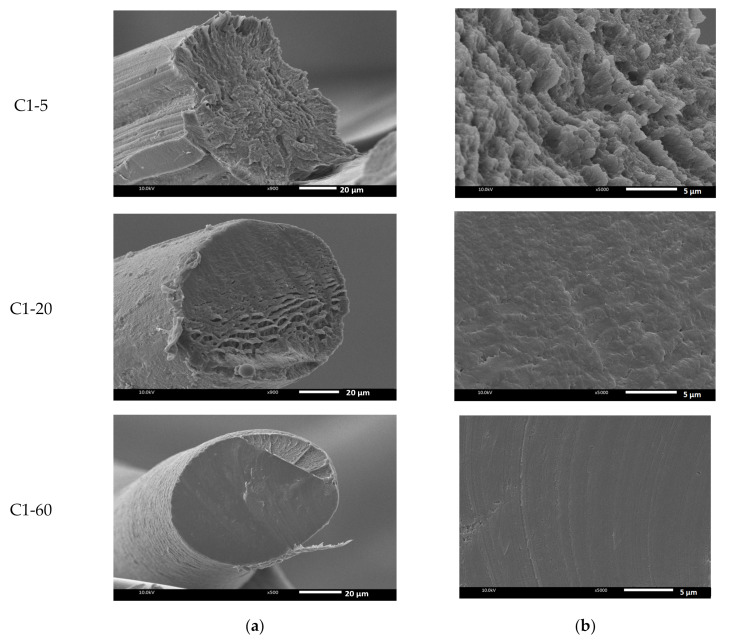
SEM images of the freeze-dried chitin fiber prepared at different temperatures of the coagulation bath at (**a**) 900× and (**b**) 5000×.

In order to investigate the differences in morphology of the fiber, Figure 4 shows the SEM images of the C1 and C2 fibers prepared at temperatures of 5, 20, and 60 °C of the coagulation bath. As shown, the morphology of the fiber structure was influenced by the coagulated temperature. In the case of C1-5, a gutter was found, as seen at a magnification of 900×. At 5 °C, the cross-sectional areas of the chitin fibers were observed with voids in different sizes (magnification of 5000×). Interestingly, when the temperature increased, the cross-sectional structure was denser, as shown in C1-20 and C1-60. In addition, the temperature highly interfered with the demixing process, leading to the formation of the ultimate fiber. The SEM photo of C2-20 at 900× magnification indicates the transformation of the cross-sectional morphology of the prepared chitin fiber. The dense and smooth cross-sectional areas were seen when the C2 fiber was produced at 60 °C. As the bath temperature increased, the fiber gained a uniform structure, and a larger number of micropores were reduced, which resulted in thicker and denser cross-sectional areas. As known, increment in temperature caused the enhanced mobility of the polymer chains, leading the higher packing of the polymer segment to influence the morphology of the chitin fiber.

**Figure 5:**
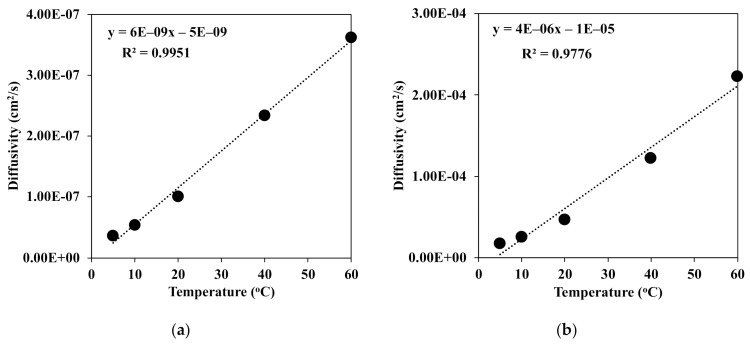
Diffusivities of (**a**) water in DMAc and (**b**) DMAc in water at different temperatures of the coagulation bath.

It was known that the exchange of the solvent and nonsolvent was one of the most important factors of the morphology of the fiber. Here, the diffusivities of water in DMAc and vice versa were measured. Figure 5 presents the diffusivity in the binary system of water and DMAc at different temperatures of the coagulation bath. With the increment of the temperature from 5 to 60 °C, the diffusivities of water and DMAc increased linearly with the correlation constant R^2^ = 0.995 for water in DMAc (Figure 5a) and 0.977 for DMAc in water (Figure 5b).

**Figure 6:**
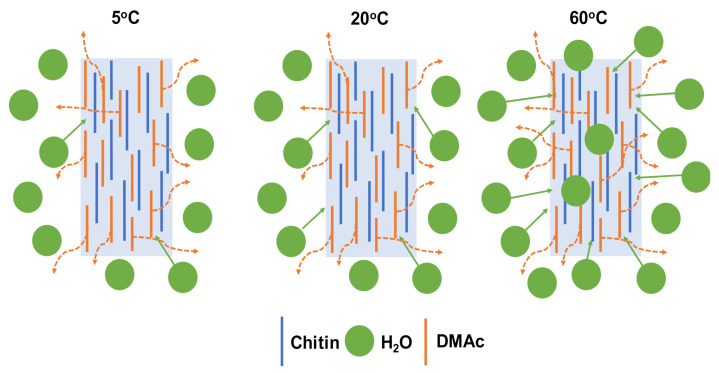
Proposed mechanism of the formation of chitin fiber at different temperatures of the water coagulation bath.

However, the diffusivity of DMAc was somehow higher than that of water at each temperature of the coagulation bath. There is no doubt that at a high temperature, the mobility of the water molecule was enhanced, which led to stronger diffusion into the chitin fiber. This means that the water molecule could much more diffuse into the coagulated chitin fiber. Then, the denser structure of the chitin fiber was rapidly obtained, as shown in Figure 6.

**Figure 7:**
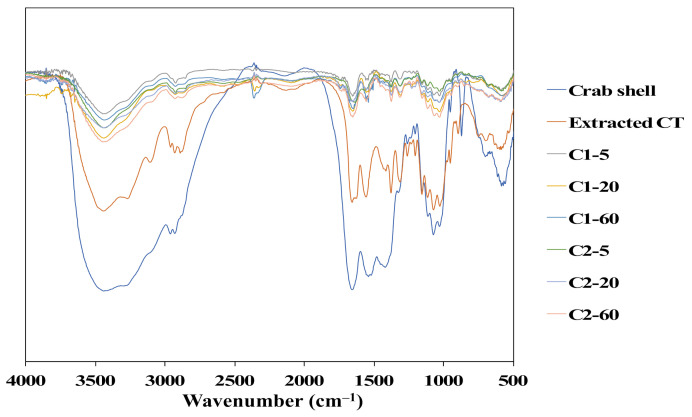
FTIR spectra of the crab shell, extracted chitin, and chitin fibers.

In the FTIR spectra of crab shell in Figure 7, the absorption peak represents calcite (CaCO_3_) at 872 cm^−1^ [25]. However, this peak disappeared after the chemical treatment, as seen in the prepared chitin. Additionally, the results show that there were specific functional groups in the purified chitin for the O–H stretching band at 3435 cm^−1^, amide I bands at 1654 cm^−1^, amides II (N–H stretching) at 3263 and 1552 cm^−1^, and C–H bonding at 2888, 2932, and 2960 cm^−1^. C–O stretching and the C–O–C ring were related at peaks of 1025 and 1155 cm^−1^, respectively. Two amide I bands observed at 1654 and 1623 cm^−1^ suggest that the chitin structure obtained was the α-form [25]. As shown, the functional groups of chitin were still well maintained in the fiber in the FTIR spectra.

**Figure 8:**
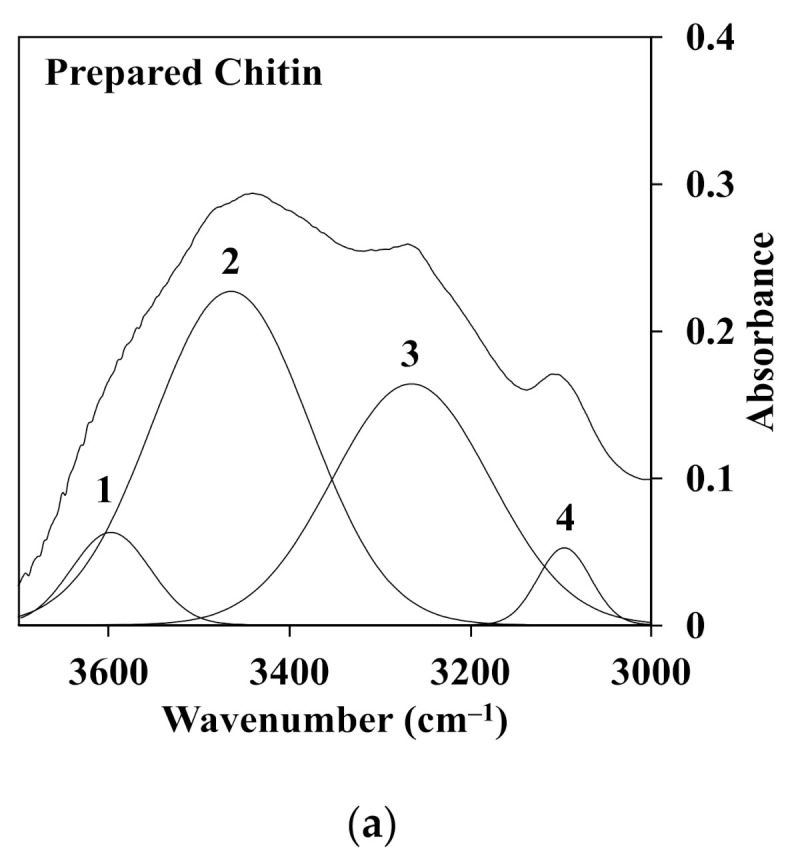

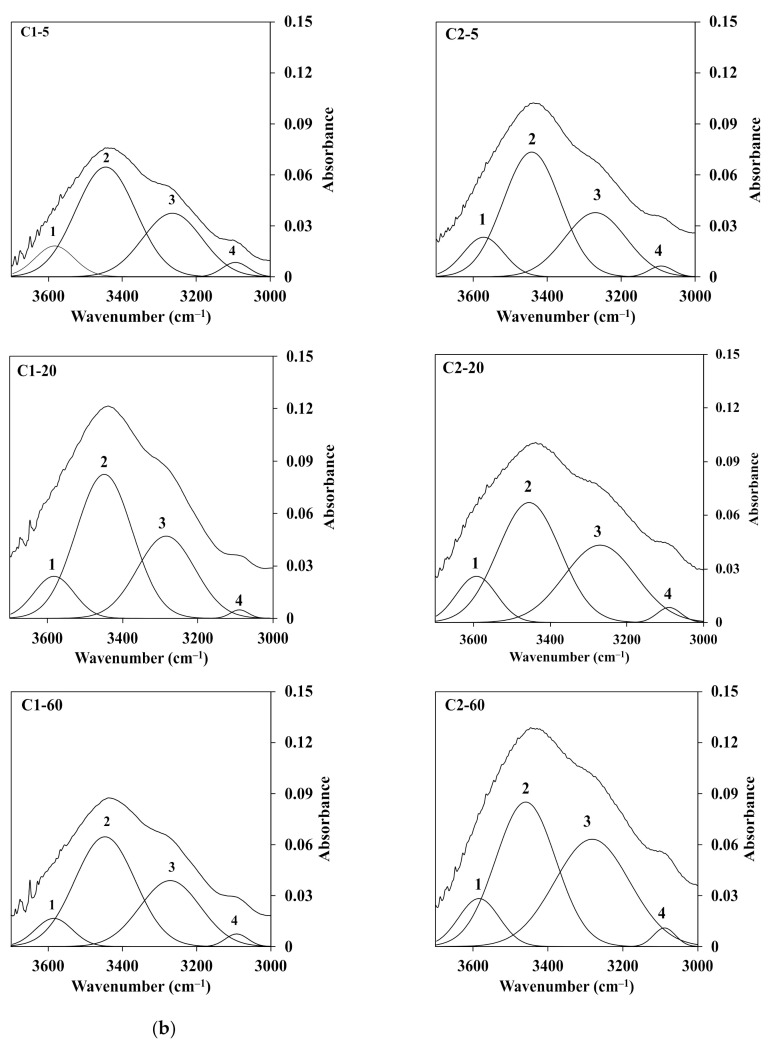
Deconvoluted FTIR spectra in the range of 3700–3000 cm^−1^ of the O–H and N–H region for the (**a**) extracted chitin and (**b**) C1.0 (left) and C2.0 (right) fiber obtained at different temperatures of the water coagulation bath.

Furthermore, the deconvolution of FTIR spectra for chitin fiber obtained at different temperatures of the water coagulation bath was carried out using a second derivative method. These spectra were decomposed into Gaussian components by curve fixed positions. Figure 8a,b shows the fixed curves of extracted chitin and C1 (left) and C2 (right) chitin fiber at temperatures of 5, 20, and 60 °C of the water coagulation bath. Normally, chitin extracted from crab shells, as shown in Figure 8a, has two intramolecular hydrogen bonds: C(6)–OH^…^O–C (peak 1) at 3597 cm^−1^ and C(3)–OH^…^O–C(5) (peak 2) at 3464 cm^−1^. In addition, the structure of α-chitin was stabilized by two intermolecular hydrogen bonds: NH^…^OC (peak 3) at 3265 cm^−1^ and C(6)–OH^…^OH–C(6) (peak 4) at 3095 cm^−1^ [27,28]. It was noted that a large portion of C(3)–OH^…^O–C(5) and NH^…^OC were the dominant bands in the hydrogen bonding networks. As observed in Figure 8b, there was a difference in the absorbance intensity of C1 (left) and C2 (right) with the increment of the temperature of the water coagulation bath. In the case of C1, the area portion of peak 3 related to the intermolecular bond of NH^…^OC was slightly changed from 31.6% to 33.4%, whereas the area portion of peak 2 was not different when the temperatures were 5 and 60 °C. Meanwhile, at a higher concentration of chitin solution as C2, the portion of peak 2 decreased, while that of peak 3 increased. For instance, with the increment of the temperature from 5 to 60 °C, the area portion of peak 2 was decreased to 55.3%, 49.2%, and 45.6% for C2-5, C2-20, and C2-60. However, the portion of peak 3 represented for the intermolecular bond of NH^…^OC was enhanced from 30.4% to 42.2% for C2-5 and C2-60. This evidence suggests that the temperature of the water coagulation bath could intensify the intermolecular interaction via hydrogen bonding of the chitin polymer chains of the fibers. This was due to the density of the chitin polymer chains in the fiber. At a higher concentration of chitin, the polymer density increased in the dense structure, which also led to the enhancement of each chitin interaction through the intermolecular hydrogen bonding. Additionally, the high temperature could intensify the molecular mobility, which enhanced the packing of the chitin segment in the fiber. Hence, both factors may conduct the packed structure of the fractured surface of the chitin fiber, as shown in the SEM images. The value of the area portion for the deconvolution of FTIR spectra is presented in Table 2.

Moreover, the polymer concentration and the temperature of the coagulant were able to influence the physical properties of the obtained fibers. Here, the results of the WC suggest that the hydrophilicity of the chitin fibers prepared at different temperatures of the water coagulation bath was affected. As described in Table 1, the values of WC for the chitin fiber decreased when the temperature of the water coagulation bath increased from 5 to 60 °C. For example, at 1% of chitin solution, the WCs of the prepared chitin fiber were 1871%, 1380%, and 725% for C1-5, C1-20, and C1-60, respectively. However, by increasing the concentration of chitin in the dope solution by up to 2%, the hydrophilicity of the chitin fiber was reduced, especially at a higher temperature. The WC was decreased from 1101% for C2-5 to 335% for C2-60. As shown in the SEM photos, the compact structure of the chitin fiber seemed to diminish the access of the water molecules absorbed. The values of WC of the prepared fiber are presented in Table 1.

As shown in Table 1, the mechanical property values of the chitin fibers were observed in a polymer-concentration- and temperature-dependent manner, which is attributed to the increment of tensile strength and elongation at the break. The results suggest that with the increment of the polymer concentration from 1% to 2%, the tensile strength of the chitin fibers obtained at 20 °C was enhanced from 82 to 104 MPa for C1-20 and C2-20. The tensile strength was much higher than that of chitin fibers prepared in trichloroacetic acid and coagulated in acetone, which had 1.03 MPa of tensile strength. However, the elongation was as high as 44% [29]. In the present study, the elongations of C1-20 and C2-20 were 17% and 19%, respectively. In the case of C2, the tensile strength and elongation of the chitin fiber prepared at 5 °C were 80 MPa and 11%. Meanwhile, the fiber obtained at a temperature of 60 °C of the coagulation bath exhibited a higher value of the mechanical property at 182 MPa with 33% extension. As shown, the elongation increased with more hydrophobic properties as obtained in the WC experiment. Another result also suggests this phenomenon in the study of Hirano [30]. However, in his case, the elongation of the chitin fiber prepared in 14% aqueous NaOH solution with a mixed coagulation solvent of H_2_SO_4_, Na_2_SO_4_, and ZnSO_4_ had a maximum elongation value of 8.4%. This could be related to the denser structure observed in SEM images at a higher temperature of the coagulation bath. As mentioned above in the deconvolution of FTIR spectra, this result could be attributed to the enhancement in the intermolecular bond of the chitin chain when the temperature of the water coagulation bath increased. This would lead to restrictions in the chain of mobility. The values of characterization of the chitin fibers are listed in Table 1.

## 4. Conclusions

In this research, chitin was chemically extracted from crab shells with the degree of acetylation at 74.6%, and the molecular weight distribution was calculated to be 5.4 × 10^5^ g/mol. The chitin fiber was prepared by phase inversion in water using the wet-spinning process. Here, the effects of polymer concentration and the temperature of the coagulation bath on the formation of the utmost chitin fiber were determined. The deconvolution of FTIR spectra indicates that the portion of the intermolecular hydrogen bonding NH^…^OC was increased with the decrease in the intrainteraction of C(3)–OH^…^O–C at a higher temperature. SEM measurement suggests that a denser structure of the fractured area was observed when the temperature of the coagulation bath increased due to the increment of the diffusivity of water molecules into coagulated chitin fiber. As a result, at a higher concentration of chitin in the dope solution, the obtained chitin fiber generated a better property in a mechanical and elastic manner. Moreover, at 2% chitin solution, the tensile strength increased from 79.6 to 182 MPa for the fiber obtained at temperatures of 5 and 60 °C of the water coagulation bath, respectively. This evidence would be appropriate for biomedical applications such as medical suture in the future due to the good mechanical strength of the fiber and the biocompatibility of the used solvent in the green synthetic route.

## Figures and Tables

**Table 1:** Characteristics of chitin solutions and fiber prepared at different temperatures of the water coagulation bath.

Temperature (°C)		Chitin Solution in DMAc/LiCl	Diameter (µm)	WC (%)	Tensile Strength (MPa)	Elongation (%)
		Shear Viscosity (Pa.s)				
**5**	C1	3.1 ± 0.3	107 ± 9	1871 ± 37	58 ± 15	8 ± 2
	C2	17.3 ± 1.6	108 ± 6	1101 ± 25	80 ± 7	11 ± 1
**20**	C1	2.0 ± 0.1	69 ± 3	1380 ± 39	82 ± 8	17 ± 4
	C2	12.6 ± 1.3	127 ± 10	1620 ± 37	104 ± 8	19 ± 1
**60**	C1	0.63 ± 0.04	71 ± 1	725 ± 29	167 ± 16	28 ± 2
	C2	3.2 ± 0.3	102 ± 2	335 ± 31	182 ± 23	33 ± 1

**Table 2:** Area portion (%) of the hydrogen bonding in the region of 3700–3000 cm^−1^ in the deconvoluted FTIR spectra.

Position	Hydrogen Bonding	% Area						
		Extracted Chitin	C1			C2		
			5 °C	20 °C	60 °C	5 °C	20 °C	60 °C
Peak 1	C(6)–OH^…^OC (intra)	7.1	3.2	1.1	2.4	2	2.4	2.2
Peak 2	C(3)–OH^…^O–C (intra)	51.3	55.2	55.3	55.5	55.3	49.2	45.6
Peak 3	NH^…^OC (inter)	37.5	31.6	32.2	33.4	30.4	36.3	42.2
Peak 4	C(6)–OH^…^OH–C(6) (inter)	4.1	10	11.4	8.7	12.3	12.1	10

## Data Availability

Not applicable.
